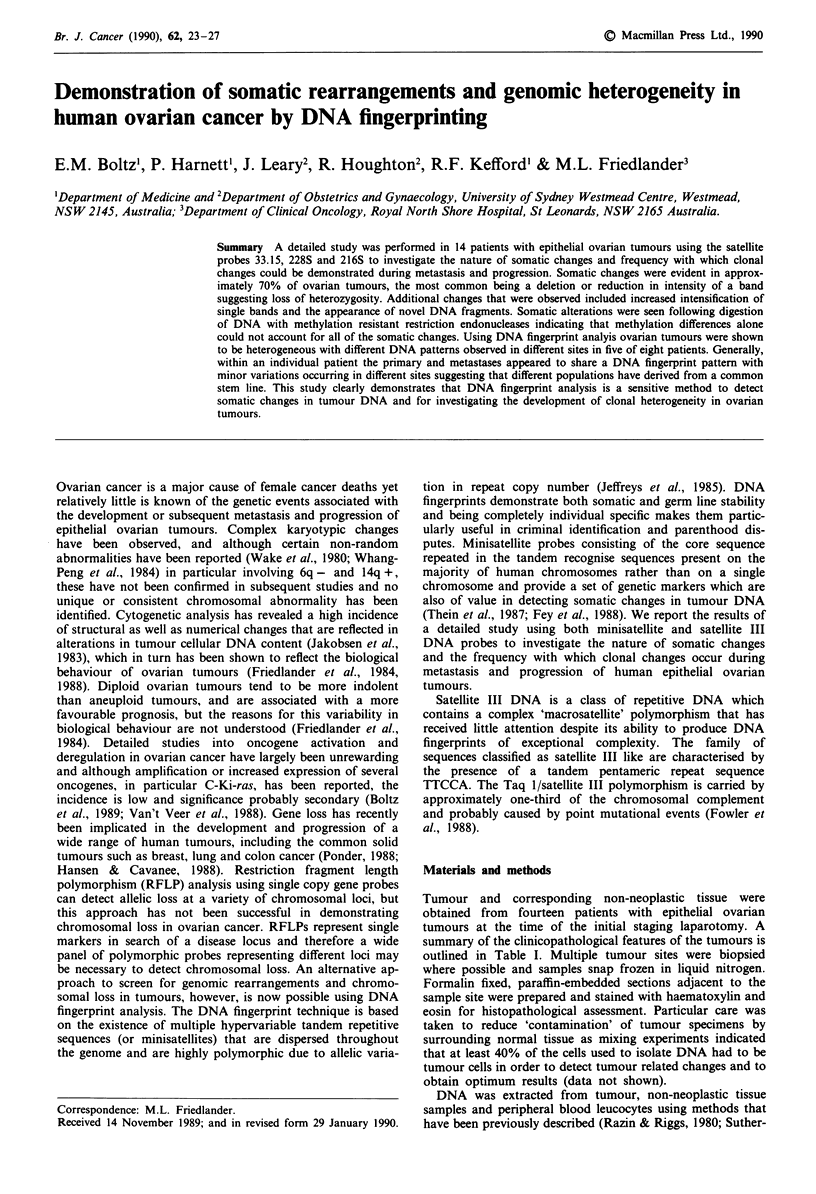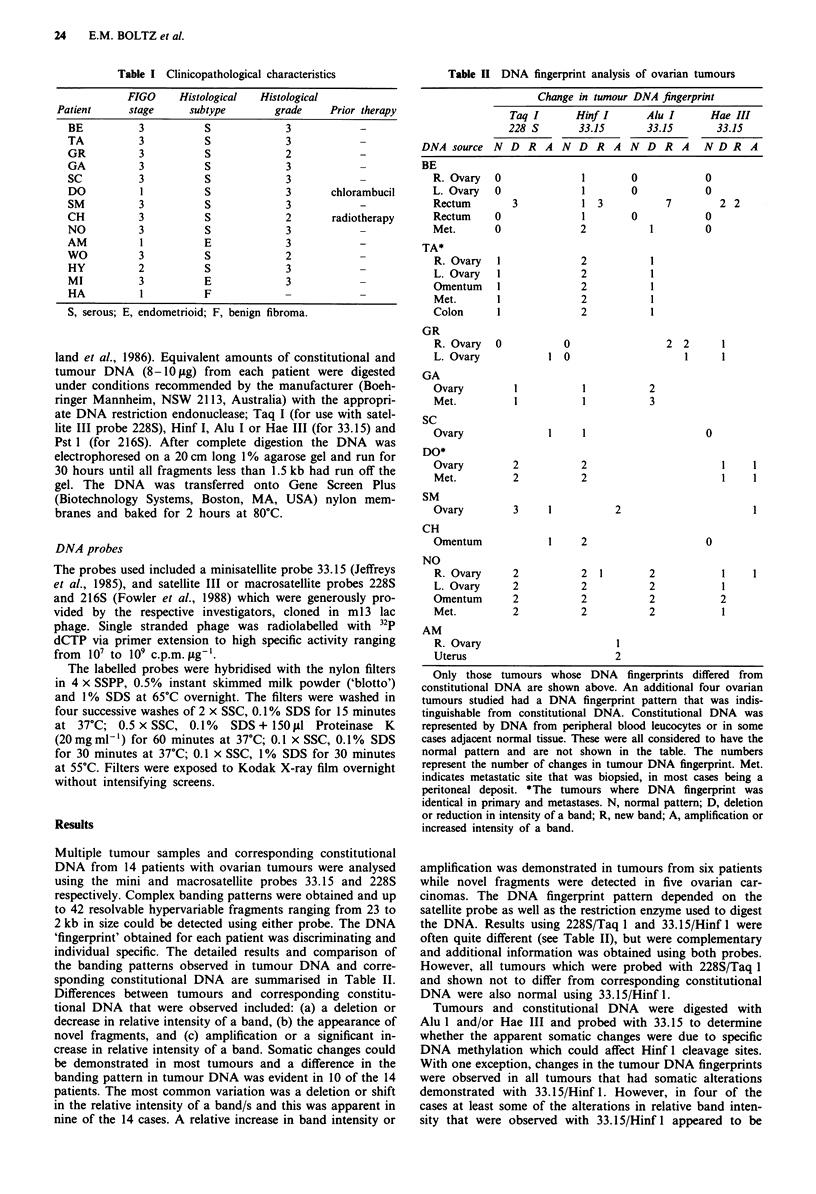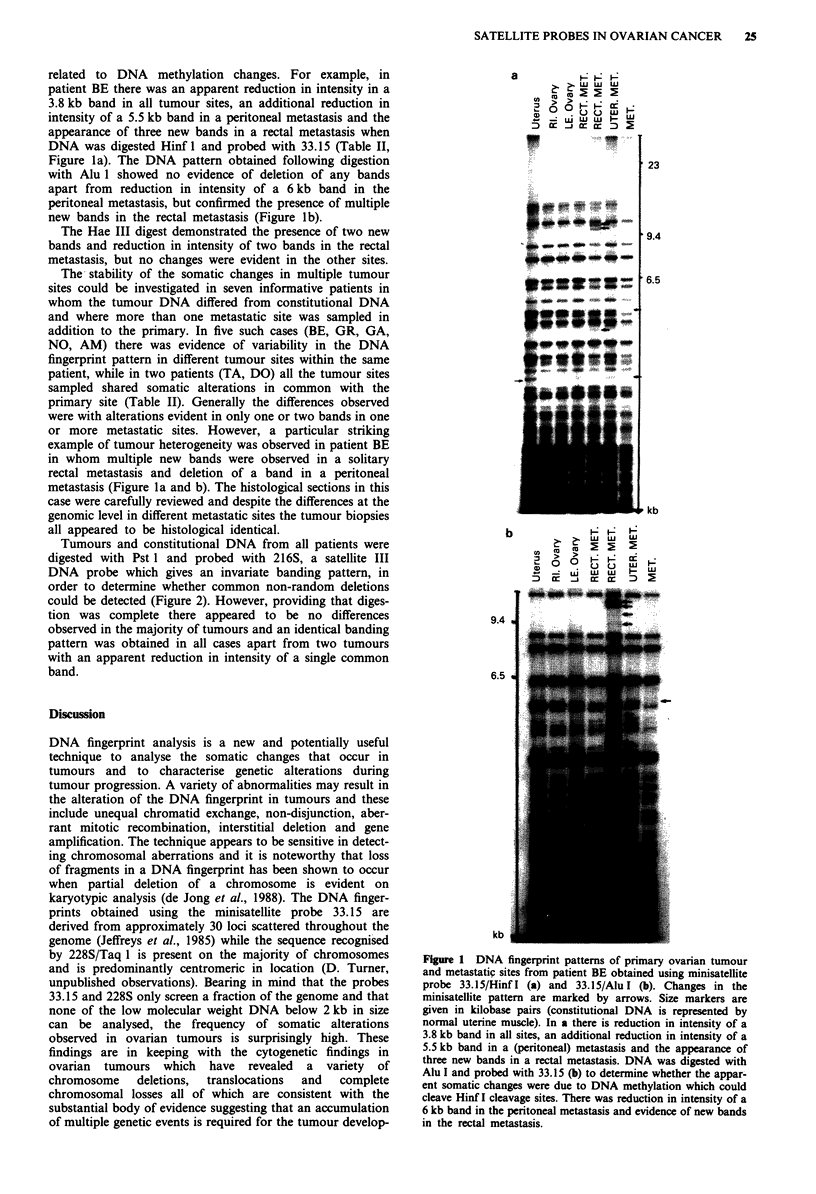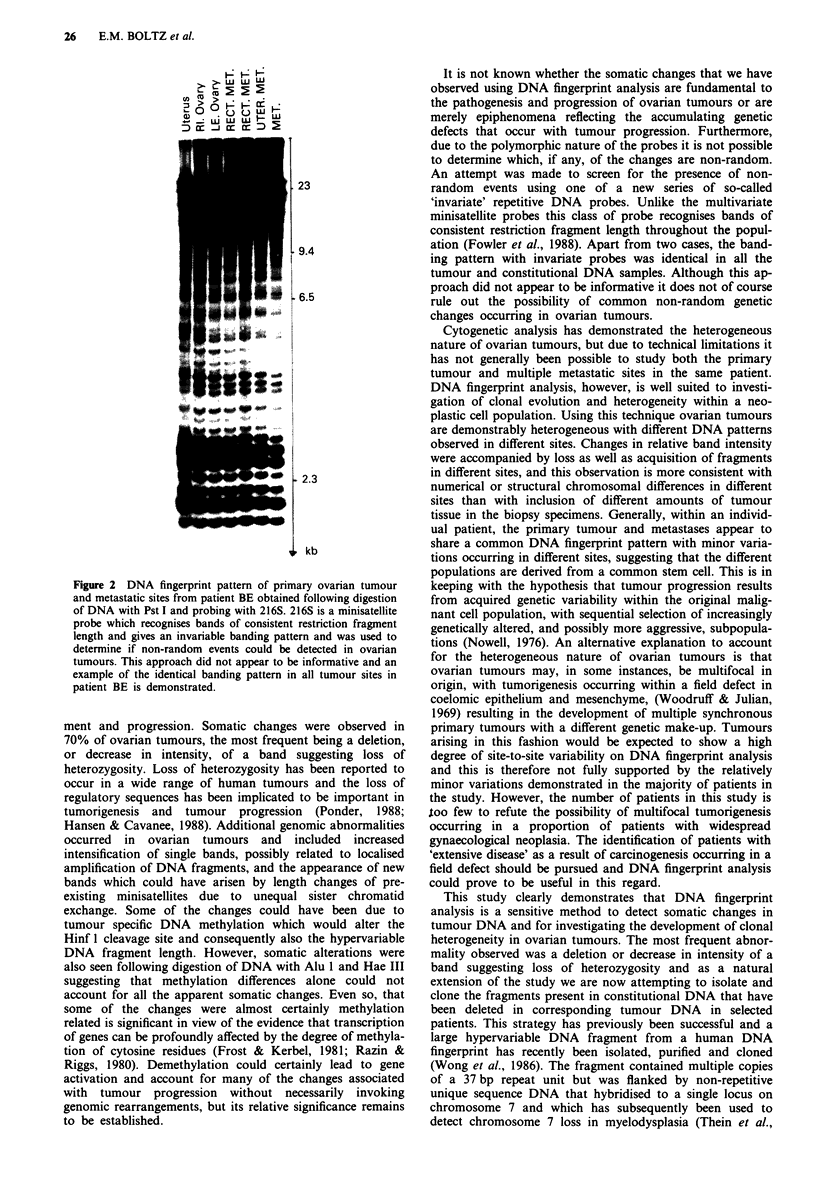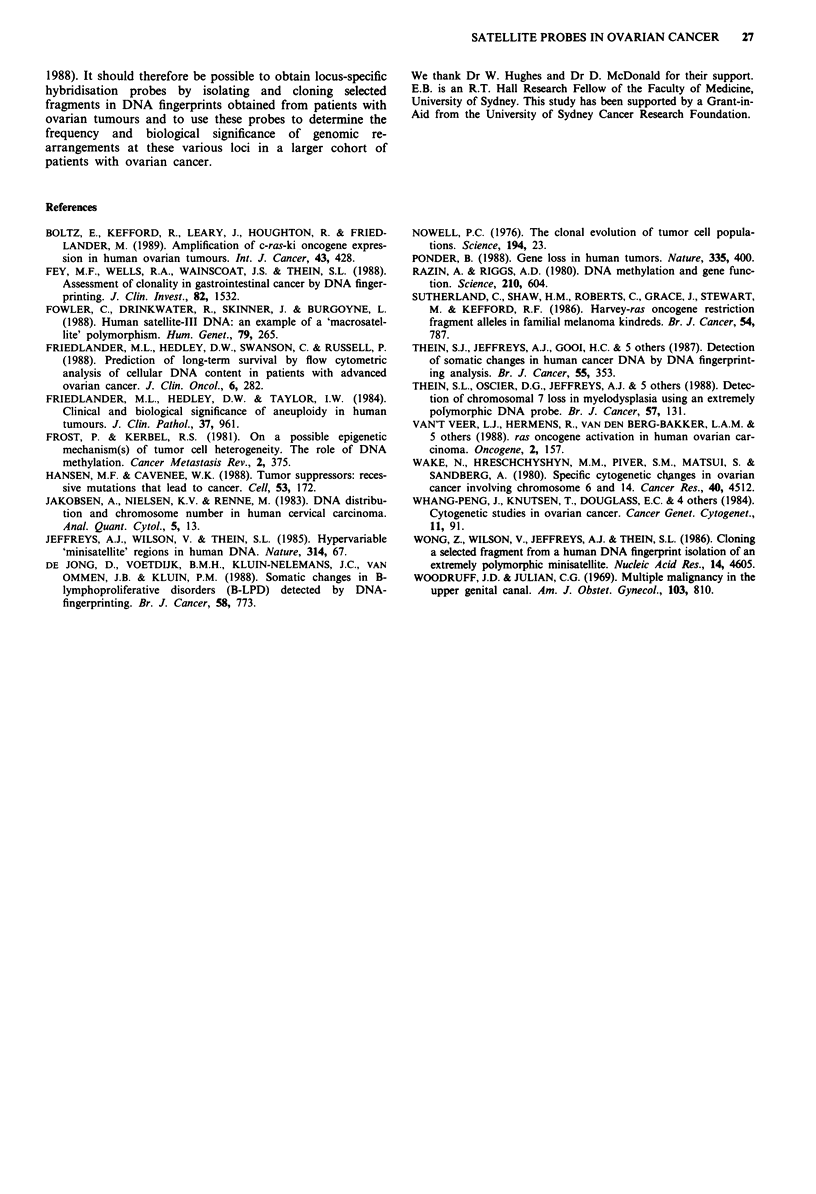# Demonstration of somatic rearrangements and genomic heterogeneity in human ovarian cancer by DNA fingerprinting.

**DOI:** 10.1038/bjc.1990.222

**Published:** 1990-07

**Authors:** E. M. Boltz, P. Harnett, J. Leary, R. Houghton, R. F. Kefford, M. L. Friedlander

**Affiliations:** Department of Medicine, University of Sydney Westmead Centre, NSW, Australia.

## Abstract

**Images:**


Br. J. Cancer (1990), 62, 23-27 © Macmillan Press Ltd., 1990

### Demonstration of somatic rearrangements and genomic heterogeneity in human ovarian cancer by DNA fingerprinting

E.M. Boltz[1], P. Harnett[1], J. Leary[2], R. Houghton[2], R.F. Kefford[1] & M.L. Friedlander[3]

[1]*Department of Medicine and* [2]*Department of Obstetrics and Gynaecology, University of Sydney Westmead Centre, Westmead, NSW 2145, Australia;* [3]*Department of Clinical Oncology, Royal North Shore Hospital, St Leonards, NSW 2165 Australia.*

**Summary** A detailed study was performed in 14 patients with epithelial ovarian tumours using the satellite probes 33.15, 228S and 216S to investigate the nature of somatic changes and frequency with which clonal changes could be demonstrated during metastasis and progression. Somatic changes were evident in approximately 70% of ovarian tumours, the most common being a deletion or reduction in intensity of a band suggesting loss of heterozygosity. Additional changes that were observed included increased intensification of single bands and the appearance of novel DNA fragments. Somatic alterations were seen following digestion of DNA with methylation resistant restriction endonucleases indicating that methylation differences alone could not account for all of the somatic changes. Using DNA fingerprint analyis ovarian tumours were shown to be heterogeneous with different DNA patterns observed in different sites in five of eight patients. Generally, within an individual patient the primary and metastases appeared to share a DNA fingerprint pattern with minor variations occurring in different sites suggesting that different populations have derived from a common stem line. This study clearly demonstrates that DNA fingerprint analysis is a sensitive method to detect somatic changes in tumour DNA and for investigating the development of clonal heterogeneity in ovarian tumours.

Ovarian cancer is a major cause of female cancer deaths yet relatively little is known of the genetic events associated with the development or subsequent metastasis and progression of epithelial ovarian tumours. Complex karyotypic changes have been observed, and although certain non-random abnormalities have been reported (Wake *et al.*, 1980; Whang-Peng *et al.*, 1984) in particular involving 6q – and 14q +, these have not been confirmed in subsequent studies and no unique or consistent chromosomal abnormality has been identified. Cytogenetic analysis has revealed a high incidence of structural as well as numerical changes that are reflected in alterations in tumour cellular DNA content (Jakobsen *et al.*, 1983), which in turn has been shown to reflect the biological behaviour of ovarian tumours (Friedlander *et al.*, 1984, 1988). Diploid ovarian tumours tend to be more indolent than aneuploid tumours, and are associated with a more favourable prognosis, but the reasons for this variability in biological behaviour are not understood (Friedlander *et al.*, 1984). Detailed studies into oncogene activation and deregulation in ovarian cancer have largely been unrewarding and although amplification or increased expression of several oncogenes, in particular C-Ki-*ras*, has been reported, the incidence is low and significance probably secondary (Boltz *et al.*, 1989; Van't Veer *et al.*, 1988). Gene loss has recently been implicated in the development and progression of a wide range of human tumours, including the common solid tumours such as breast, lung and colon cancer (Ponder, 1988; Hansen & Cavanee, 1988). Restriction fragment length polymorphism (RFLP) analysis using single copy gene probes can detect allelic loss at a variety of chromosomal loci, but this approach has not been successful in demonstrating chromosomal loss in ovarian cancer. RFLPs represent single markers in search of a disease locus and therefore a wide panel of polymorphic probes representing different loci may be necessary to detect chromosomal loss. An alternative approach to screen for genomic rearrangements and chromosomal loss in tumours, however, is now possible using DNA fingerprint analysis. The DNA fingerprint technique is based on the existence of multiple hypervariable tandem repetitive sequences (or minisatellites) that are dispersed throughout the genome and are highly polymorphic due to allelic variation in repeat copy number (Jeffreys *et al.*, 1985). DNA fingerprints demonstrate both somatic and germ line stability and being completely individual specific makes them particularly useful in criminal identification and parenthood disputes. Minisatellite probes consisting of the core sequence repeated in the tandem recognise sequences present on the majority of human chromosomes rather than on a single chromosome and provide a set of genetic markers which are also of value in detecting somatic changes in tumour DNA (Thein *et al.*, 1987; Fey *et al.*, 1988). We report the results of a detailed study using both minisatellite and satellite III DNA probes to investigate the nature of somatic changes and the frequency with which clonal changes occur during metastasis and progression of human epithelial ovarian tumours.

Satellite III DNA is a class of repetitive DNA which contains a complex 'macrosatellite' polymorphism that has received little attention despite its ability to produce DNA fingerprints of exceptional complexity. The family of sequences classified as satellite III like are characterised by the presence of a tandem pentameric repeat sequence TTCCA. The Taq 1/satellite III polymorphism is carried by approximately one-third of the chromosomal complement and probably caused by point mutational events (Fowler *et al.*, 1988).

### Materials and methods

Tumour and corresponding non-neoplastic tissue were obtained from fourteen patients with epithelial ovarian tumours at the time of the initial staging laparotomy. A summary of the clinicopathological features of the tumours is outlined in Table I. Multiple tumour sites were biopsied where possible and samples snap frozen in liquid nitrogen. Formalin fixed, paraffin-embedded sections adjacent to the sample site were prepared and stained with haematoxylin and eosin for histopathological assessment. Particular care was taken to reduce 'contamination' of tumour specimens by surrounding normal tissue as mixing experiments indicated that at least 40% of the cells used to isolate DNA had to be tumour cells in order to detect tumour related changes and to obtain optimum results (data not shown).

DNA was extracted from tumour, non-neoplastic tissue samples and peripheral blood leucocytes using methods that have been previously described (Razin & Riggs, 1980; Suther-

Correspondence: M.L. Friedlander.
Received 14 November 1989; and in revised form 29 January 1990.

**Table I** Clinicopathological characteristics

| Patient | *FIGO stage* | *Histological subtype* | *Histological grade* | *Prior therapy* |
|---|---|---|---|---|
| BE | 3 | S | 3 | – |
| TA | 3 | S | 3 | – |
| GR | 3 | S | 2 | – |
| GA | 3 | S | 3 | – |
| SC | 3 | S | 3 | – |
| DO | 1 | S | 3 | chlorambucil |
| SM | 3 | S | 3 | – |
| CH | 3 | S | 2 | radiotherapy |
| NO | 3 | S | 3 | – |
| AM | 1 | E | 3 | – |
| WO | 3 | S | 2 | – |
| HY | 2 | S | 3 | – |
| MI | 3 | E | 3 | – |
| HA | 1 | F | – | – |

S, serous; E, endometrioid; F, benign fibroma.

land *et al.*, 1986). Equivalent amounts of constitutional and tumour DNA (8–10 μg) from each patient were digested under conditions recommended by the manufacturer (Boehringer Mannheim, NSW 2113, Australia) with the appropriate DNA restriction endonuclease; Taq I (for use with satellite III probe 228S), Hinf I, Alu I or Hae III (for 33.15) and Pst 1 (for 216S). After complete digestion the DNA was electrophoresed on a 20 cm long 1% agarose gel and run for 30 hours until all fragments less than 1.5 kb had run off the gel. The DNA was transferred onto Gene Screen Plus (Biotechnology Systems, Boston, MA, USA) nylon membranes and baked for 2 hours at 80°C.

### *DNA probes*

The probes used included a minisatellite probe 33.15 (Jeffreys *et al.*, 1985), and satellite III or macrosatellite probes 228S and 216S (Fowler *et al.*, 1988) which were generously provided by the respective investigators, cloned in m13 lac phage. Single stranded phage was radiolabelled with $^{32}P$ dCTP via primer extension to high specific activity ranging from $10^7$ to $10^9$ c.p.m. $\mu g^{-1}$.

The labelled probes were hybridised with the nylon filters in 4 × SSPP, 0.5% instant skimmed milk powder ('blotto') and 1% SDS at 65°C overnight. The filters were washed in four successive washes of 2 × SSC, 0.1% SDS for 15 minutes at 37°C; 0.5 × SSC, 0.1% SDS + 150 μl Proteinase K (20 mg $ml^{-1}$) for 60 minutes at 37°C; 0.1 × SSC, 0.1% SDS for 30 minutes at 37°C; 0.1 × SSC, 1% SDS for 30 minutes at 55°C. Filters were exposed to Kodak X-ray film overnight without intensifying screens.

### Results

Multiple tumour samples and corresponding constitutional DNA from 14 patients with ovarian tumours were analysed using the mini and macrosatellite probes 33.15 and 228S respectively. Complex banding patterns were obtained and up to 42 resolvable hypervariable fragments ranging from 23 to 2 kb in size could be detected using either probe. The DNA 'fingerprint' obtained for each patient was discriminating and individual specific. The detailed results and comparison of the banding patterns observed in tumour DNA and corresponding constitutional DNA are summarised in Table II. Differences between tumours and corresponding constitutional DNA that were observed included: (a) a deletion or decrease in relative intensity of a band, (b) the appearance of novel fragments, and (c) amplification or a significant increase in relative intensity of a band. Somatic changes could be demonstrated in most tumours and a difference in the banding pattern in tumour DNA was evident in 10 of the 14 patients. The most common variation was a deletion or shift in the relative intensity of a band/s and this was apparent in nine of the 14 cases. A relative increase in band intensity or amplification was demonstrated in tumours from six patients while novel fragments were detected in five ovarian carcinomas. The DNA fingerprint pattern depended on the satellite probe as well as the restriction enzyme used to digest the DNA. Results using 228S/Taq 1 and 33.15/Hinf 1 were often quite different (see Table II), but were complementary and additional information was obtained using both probes. However, all tumours which were probed with 228S/Taq 1 and shown not to differ from corresponding constitutional DNA were also normal using 33.15/Hinf 1.

Tumours and constitutional DNA were digested with Alu 1 and/or Hae III and probed with 33.15 to determine whether the apparent somatic changes were due to specific DNA methylation which could affect Hinf 1 cleavage sites. With one exception, changes in the tumour DNA fingerprints were observed in all tumours that had somatic alterations demonstrated with 33.15/Hinf 1. However, in four of the cases at least some of the alterations in relative band intensity that were observed with 33.15/Hinf 1 appeared to be

**Table II** DNA fingerprint analysis of ovarian tumours

| | *Change in tumour DNA fingerprint* | | | | | | | | | | | | | | | |
|---|---|---|---|---|---|---|---|---|---|---|---|---|---|---|---|---|
| | *Taq I 228 S* | | | | *Hinf I 33.15* | | | | *Alu I 33.15* | | | | *Hae III 33.15* | | | |
| *DNA source* | *N* | *D* | *R* | *A* | *N* | *D* | *R* | *A* | *N* | *D* | *R* | *A* | *N* | *D* | *R* | *A* |
| BE | | | | | | | | | | | | | | | | |
| R. Ovary | 0 | | | | | 1 | | | 0 | | | | 0 | | | |
| L. Ovary | 0 | | | | | 1 | | | 0 | | | | 0 | | | |
| Rectum | | 3 | | | | 1 | 3 | | | | 7 | | | 2 | 2 | |
| Rectum | 0 | | | | | 1 | | | 0 | | | | 0 | | | |
| Met. | 0 | | | | | 2 | | | | 1 | | | 0 | | | |
| TA* | | | | | | | | | | | | | | | | |
| R. Ovary | 1 | | | | | 2 | | | | 1 | | | | | | |
| L. Ovary | 1 | | | | | 2 | | | | 1 | | | | | | |
| Omentum | 1 | | | | | 2 | | | | 1 | | | | | | |
| Met. | 1 | | | | | 2 | | | | 1 | | | | | | |
| Colon | 1 | | | | | 2 | | | | 1 | | | | | | |
| GR | | | | | | | | | | | | | | | | |
| R. Ovary | 0 | | | | 0 | | | | | | 2 | 2 | | 1 | | |
| L. Ovary | | | | 1 | 0 | | | | | | | 1 | | 1 | | |
| GA | | | | | | | | | | | | | | | | |
| Ovary | | 1 | | | | 1 | | | | 2 | | | | | | |
| Met. | | 1 | | | | 1 | | | | 3 | | | | | | |
| SC | | | | | | | | | | | | | | | | |
| Ovary | | | | 1 | | 1 | | | | | | | 0 | | | |
| DO* | | | | | | | | | | | | | | | | |
| Ovary | | 2 | | | | 2 | | | | | | | | 1 | | 1 |
| Met. | | 2 | | | | 2 | | | | | | | | 1 | | 1 |
| SM | | | | | | | | | | | | | | | | |
| Ovary | | 3 | | 1 | | | | 2 | | | | | | | | 1 |
| CH | | | | | | | | | | | | | | | | |
| Omentum | | | | 1 | | 2 | | | | | | | 0 | | | |
| NO | | | | | | | | | | | | | | | | |
| R. Ovary | | 2 | | | | 2 | 1 | | | 2 | | | | 1 | | 1 |
| L. Ovary | | 2 | | | | 2 | | | | 2 | | | | 1 | | |
| Omentum | | 2 | | | | 2 | | | | 2 | | | | 2 | | |
| Met. | | 2 | | | | 2 | | | | 2 | | | | 1 | | |
| AM | | | | | | | | | | | | | | | | |
| R. Ovary | | | | | | | | 1 | | | | | | | | |
| Uterus | | | | | | | | 2 | | | | | | | | |

Only those tumours whose DNA fingerprints differed from constitutional DNA are shown above. An additional four ovarian tumours studied had a DNA fingerprint pattern that was indistinguishable from constitutional DNA. Constitutional DNA was represented by DNA from peripheral blood leucocytes or in some cases adjacent normal tissue. These were all considered to have the normal pattern and are not shown in the table. The numbers represent the number of changes in tumour DNA fingerprint. Met. indicates metastatic site that was biopsied, in most cases being a peritoneal deposit. *The tumours where DNA fingerprint was identical in primary and metastases. N, normal pattern; D, deletion or reduction in intensity of a band; R, new band; A, amplification or increased intensity of a band.

related to DNA methylation changes. For example, in patient BE there was an apparent reduction in intensity in a 3.8 kb band in all tumour sites, an additional reduction in intensity of a 5.5 kb band in a peritoneal metastasis and the appearance of three new bands in a rectal metastasis when DNA was digested Hinf 1 and probed with 33.15 (Table II, Figure 1a). The DNA pattern obtained following digestion with Alu 1 showed no evidence of deletion of any bands apart from reduction in intensity of a 6 kb band in the peritoneal metastasis, but confirmed the presence of multiple new bands in the rectal metastasis (Figure 1b).

The Hae III digest demonstrated the presence of two new bands and reduction in intensity of two bands in the rectal metastasis, but no changes were evident in the other sites.

The stability of the somatic changes in multiple tumour sites could be investigated in seven informative patients in whom the tumour DNA differed from constitutional DNA and where more than one metastatic site was sampled in addition to the primary. In five such cases (BE, GR, GA, NO, AM) there was evidence of variability in the DNA fingerprint pattern in different tumour sites within the same patient, while in two patients (TA, DO) all the tumour sites sampled shared somatic alterations in common with the primary site (Table II). Generally the differences observed were with alterations evident in only one or two bands in one or more metastatic sites. However, a particular striking example of tumour heterogeneity was observed in patient BE in whom multiple new bands were observed in a solitary rectal metastasis and deletion of a band in a peritoneal metastasis (Figure 1a and b). The histological sections in this case were carefully reviewed and despite the differences at the genomic level in different metastatic sites the tumour biopsies all appeared to be histological identical.

Tumours and constitutional DNA from all patients were digested with Pst 1 and probed with 216S, a satellite III DNA probe which gives an invariate banding pattern, in order to determine whether common non-random deletions could be detected (Figure 2). However, providing that digestion was complete there appeared to be no differences observed in the majority of tumours and an identical banding pattern was obtained in all cases apart from two tumours with an apparent reduction in intensity of a single common band.

### Discussion

DNA fingerprint analysis is a new and potentially useful technique to analyse the somatic changes that occur in tumours and to characterise genetic alterations during tumour progression. A variety of abnormalities may result in the alteration of the DNA fingerprint in tumours and these include unequal chromatid exchange, non-disjunction, aberrant mitotic recombination, interstitial deletion and gene amplification. The technique appears to be sensitive in detecting chromosomal aberrations and it is noteworthy that loss of fragments in a DNA fingerprint has been shown to occur when partial deletion of a chromosome is evident on karyotypic analysis (de Jong *et al.*, 1988). The DNA fingerprints obtained using the minisatellite probe 33.15 are derived from approximately 30 loci scattered throughout the genome (Jeffreys *et al.*, 1985) while the sequence recognised by 228S/Taq 1 is present on the majority of chromosomes and is predominantly centromeric in location (D. Turner, unpublished observations). Bearing in mind that the probes 33.15 and 228S only screen a fraction of the genome and that none of the low molecular weight DNA below 2 kb in size can be analysed, the frequency of somatic alterations observed in ovarian tumours is surprisingly high. These findings are in keeping with the cytogenetic findings in ovarian tumours which have revealed a variety of chromosome deletions, translocations and complete chromosomal losses all of which are consistent with the substantial body of evidence suggesting that an accumulation of multiple genetic events is required for the tumour develop-

**Figure 1** DNA fingerprint patterns of primary ovarian tumour and metastatic sites from patient BE obtained using minisatellite probe 33.15/Hinf I (**a**) and 33.15/Alu I (**b**). Changes in the minisatellite pattern are marked by arrows. Size markers are given in kilobase pairs (constitutional DNA is represented by normal uterine muscle). In **a** there is reduction in intensity of a 3.8 kb band in all sites, an additional reduction in intensity of a 5.5 kb band in a (peritoneal) metastasis and the appearance of three new bands in a rectal metastasis. DNA was digested with Alu I and probed with 33.15 (**b**) to determine whether the apparent somatic changes were due to DNA methylation which could cleave Hinf I cleavage sites. There was reduction in intensity of a 6 kb band in the peritoneal metastasis and evidence of new bands in the rectal metastasis.

**Figure 2** DNA fingerprint pattern of primary ovarian tumour and metastatic sites from patient BE obtained following digestion of DNA with Pst I and probing with 216S. 216S is a minisatellite probe which recognises bands of consistent restriction fragment length and gives an invariable banding pattern and was used to determine if non-random events could be detected in ovarian tumours. This approach did not appear to be informative and an example of the identical banding pattern in all tumour sites in patient BE is demonstrated.

ment and progression. Somatic changes were observed in 70% of ovarian tumours, the most frequent being a deletion, or decrease in intensity, of a band suggesting loss of heterozygosity. Loss of heterozygosity has been reported to occur in a wide range of human tumours and the loss of regulatory sequences has been implicated to be important in tumorigenesis and tumour progression (Ponder, 1988; Hansen & Cavanee, 1988). Additional genomic abnormalities occurred in ovarian tumours and included increased intensification of single bands, possibly related to localised amplification of DNA fragments, and the appearance of new bands which could have arisen by length changes of pre-existing minisatellites due to unequal sister chromatid exchange. Some of the changes could have been due to tumour specific DNA methylation which would alter the Hinf 1 cleavage site and consequently also the hypervariable DNA fragment length. However, somatic alterations were also seen following digestion of DNA with Alu 1 and Hae III suggesting that methylation differences alone could not account for all the apparent somatic changes. Even so, that some of the changes were almost certainly methylation related is significant in view of the evidence that transcription of genes can be profoundly affected by the degree of methylation of cytosine residues (Frost & Kerbel, 1981; Razin & Riggs, 1980). Demethylation could certainly lead to gene activation and account for many of the changes associated with tumour progression without necessarily invoking genomic rearrangements, but its relative significance remains to be established.

It is not known whether the somatic changes that we have observed using DNA fingerprint analysis are fundamental to the pathogenesis and progression of ovarian tumours or are merely epiphenomena reflecting the accumulating genetic defects that occur with tumour progression. Furthermore, due to the polymorphic nature of the probes it is not possible to determine which, if any, of the changes are non-random. An attempt was made to screen for the presence of non-random events using one of a new series of so-called 'invariate' repetitive DNA probes. Unlike the multivariate minisatellite probes this class of probe recognises bands of consistent restriction fragment length throughout the population (Fowler et al., 1988). Apart from two cases, the banding pattern with invariate probes was identical in all the tumour and constitutional DNA samples. Although this approach did not appear to be informative it does not of course rule out the possibility of common non-random genetic changes occurring in ovarian tumours.

Cytogenetic analysis has demonstrated the heterogeneous nature of ovarian tumours, but due to technical limitations it has not generally been possible to study both the primary tumour and multiple metastatic sites in the same patient. DNA fingerprint analysis, however, is well suited to investigation of clonal evolution and heterogeneity within a neoplastic cell population. Using this technique ovarian tumours are demonstrably heterogeneous with different DNA patterns observed in different sites. Changes in relative band intensity were accompanied by loss as well as acquisition of fragments in different sites, and this observation is more consistent with numerical or structural chromosomal differences in different sites than with inclusion of different amounts of tumour tissue in the biopsy specimens. Generally, within an individual patient, the primary tumour and metastases appear to share a common DNA fingerprint pattern with minor variations occurring in different sites, suggesting that the different populations are derived from a common stem cell. This is in keeping with the hypothesis that tumour progression results from acquired genetic variability within the original malignant cell population, with sequential selection of increasingly genetically altered, and possibly more aggressive, subpopulations (Nowell, 1976). An alternative explanation to account for the heterogeneous nature of ovarian tumours is that ovarian tumours may, in some instances, be multifocal in origin, with tumorigenesis occurring within a field defect in coelomic epithelium and mesenchyme, (Woodruff & Julian, 1969) resulting in the development of multiple synchronous primary tumours with a different genetic make-up. Tumours arising in this fashion would be expected to show a high degree of site-to-site variability on DNA fingerprint analysis and this is therefore not fully supported by the relatively minor variations demonstrated in the majority of patients in the study. However, the number of patients in this study is too few to refute the possibility of multifocal tumorigenesis occurring in a proportion of patients with widespread gynaecological neoplasia. The identification of patients with 'extensive disease' as a result of carcinogenesis occurring in a field defect should be pursued and DNA fingerprint analysis could prove to be useful in this regard.

This study clearly demonstrates that DNA fingerprint analysis is a sensitive method to detect somatic changes in tumour DNA and for investigating the development of clonal heterogeneity in ovarian tumours. The most frequent abnormality observed was a deletion or decrease in intensity of a band suggesting loss of heterozygosity and as a natural extension of the study we are now attempting to isolate and clone the fragments present in constitutional DNA that have been deleted in corresponding tumour DNA in selected patients. This strategy has previously been successful and a large hypervariable DNA fragment from a human DNA fingerprint has recently been isolated, purified and cloned (Wong et al., 1986). The fragment contained multiple copies of a 37 bp repeat unit but was flanked by non-repetitive unique sequence DNA that hybridised to a single locus on chromosome 7 and which has subsequently been used to detect chromosome 7 loss in myelodysplasia (Thein et al.,

1988). It should therefore be possible to obtain locus-specific hybridisation probes by isolating and cloning selected fragments in DNA fingerprints obtained from patients with ovarian tumours and to use these probes to determine the frequency and biological significance of genomic rearrangements at these various loci in a larger cohort of patients with ovarian cancer.

We thank Dr W. Hughes and Dr D. McDonald for their support. E.B. is an R.T. Hall Research Fellow of the Faculty of Medicine, University of Sydney. This study has been supported by a Grant-in-Aid from the University of Sydney Cancer Research Foundation.

### References

BOLTZ, E., KEFFORD, R., LEARY, J., HOUGHTON, R. & FRIEDLANDER, M. (1989). Amplification of c-*ras*-ki oncogene expression in human ovarian tumours. *Int. J. Cancer*, **43**, 428.

FEY, M.F., WELLS, R.A., WAINSCOAT, J.S. & THEIN, S.L. (1988). Assessment of clonality in gastrointestinal cancer by DNA fingerprinting. *J. Clin. Invest.*, **82**, 1532.

FOWLER, C., DRINKWATER, R., SKINNER, J. & BURGOYNE, L. (1988). Human satellite-III DNA: an example of a 'macrosatellite' polymorphism. *Hum. Genet.*, **79**, 265.

FRIEDLANDER, M.L., HEDLEY, D.W., SWANSON, C. & RUSSELL, P. (1988). Prediction of long-term survival by flow cytometric analysis of cellular DNA content in patients with advanced ovarian cancer. *J. Clin. Oncol.*, **6**, 282.

FRIEDLANDER, M.L., HEDLEY, D.W. & TAYLOR, I.W. (1984). Clinical and biological significance of aneuploidy in human tumours. *J. Clin. Pathol.*, **37**, 961.

FROST, P. & KERBEL, R.S. (1981). On a possible epigenetic mechanism(s) of tumor cell heterogeneity. The role of DNA methylation. *Cancer Metastasis Rev.*, **2**, 375.

HANSEN, M.F. & CAVENEE, W.K. (1988). Tumor suppressors: recessive mutations that lead to cancer. *Cell*, **53**, 172.

JAKOBSEN, A., NIELSEN, K.V. & RENNE, M. (1983). DNA distribution and chromosome number in human cervical carcinoma. *Anal. Quant. Cytol.*, **5**, 13.

JEFFREYS, A.J., WILSON, V. & THEIN, S.L. (1985). Hypervariable 'minisatellite' regions in human DNA. *Nature*, **314**, 67.

DE JONG, D., VOETDIJK, B.M.H., KLUIN-NELEMANS, J.C., VAN OMMEN, J.B. & KLUIN, P.M. (1988). Somatic changes in B-lymphoproliferative disorders (B-LPD) detected by DNA-fingerprinting. *Br. J. Cancer*, **58**, 773.

NOWELL, P.C. (1976). The clonal evolution of tumor cell populations. *Science*, **194**, 23.

PONDER, B. (1988). Gene loss in human tumors. *Nature*, **335**, 400.

RAZIN, A. & RIGGS, A.D. (1980). DNA methylation and gene function. *Science*, **210**, 604.

SUTHERLAND, C., SHAW, H.M., ROBERTS, C., GRACE, J., STEWART, M. & KEFFORD, R.F. (1986). Harvey-*ras* oncogene restriction fragment alleles in familial melanoma kindreds. *Br. J. Cancer*, **54**, 787.

THEIN, S.J., JEFFREYS, A.J., GOOI, H.C. & 5 others (1987). Detection of somatic changes in human cancer DNA by DNA fingerprinting analysis. *Br. J. Cancer*, **55**, 353.

THEIN, S.L., OSCIER, D.G., JEFFREYS, A.J. & 5 others (1988). Detection of chromosomal 7 loss in myelodysplasia using an extremely polymorphic DNA probe. *Br. J. Cancer*, **57**, 131.

VAN'T VEER, L.J., HERMENS, R., VAN DEN BERG-BAKKER, L.A.M. & 5 others (1988). *ras* oncogene activation in human ovarian carcinoma. *Oncogene*, **2**, 157.

WAKE, N., HRESCHCHYSHYN, M.M., PIVER, S.M., MATSUI, S. & SANDBERG, A. (1980). Specific cytogenetic changes in ovarian cancer involving chromosome 6 and 14. *Cancer Res.*, **40**, 4512.

WHANG-PENG, J., KNUTSEN, T., DOUGLASS, E.C. & 4 others (1984). Cytogenetic studies in ovarian cancer. *Cancer Genet. Cytogenet.*, **11**, 91.

WONG, Z., WILSON, V., JEFFREYS, A.J. & THEIN, S.L. (1986). Cloning a selected fragment from a human DNA fingerprint isolation of an extremely polymorphic minisatellite. *Nucleic Acid Res.*, **14**, 4605.

WOODRUFF, J.D. & JULIAN, C.G. (1969). Multiple malignancy in the upper genital canal. *Am. J. Obstet. Gynecol.*, **103**, 810.